# Immune and Viral Correlates of “Secondary Viral Control” after Treatment Interruption in Chronically HIV-1 Infected Patients

**DOI:** 10.1371/journal.pone.0037792

**Published:** 2012-05-30

**Authors:** Ellen Van Gulck, Lotte Bracke, Leo Heyndrickx, Sandra Coppens, Derek Atkinson, Céline Merlin, Alexander Pasternak, Eric Florence, Guido Vanham

**Affiliations:** 1 Virology Unit, Microbiology Group, Department of Biomedical Sciences, Institute of Tropical Medicine, Antwerp (ITMA), Antwerp, Belgium; 2 Laboratory of Experimental Virology, Department of Medical Microbiology, Center for Infection and Immunity Amsterdam (CINIMA), Amsterdam, The Netherlands; 3 HIV/STD Unit, Department of Clinical Sciences, ITMA, Antwerp, Belgium; 4 Department of Biomedical Sciences, Faculty of Pharmaceutical, Veterinary and Biomedical Sciences, University of Antwerp, Antwerp, Belgium; University of Alabama, United States of America

## Abstract

Upon interruption of antiretroviral therapy, HIV-infected patients usually show viral load rebound to pre-treatment levels. Four patients, hereafter referred to as secondary controllers (SC), were identified who initiated therapy during chronic infection and, after stopping treatment, could control virus replication at undetectable levels for more than six months. In the present study we set out to unravel possible viral and immune parameters or mechanisms of this phenomenon by comparing secondary controllers with elite controllers and non-controllers, including patients under HAART. As candidate correlates of protection, virus growth kinetics, levels of intracellular viral markers, several aspects of HIV-specific CD4+ and CD8+ T cell function and HIV neutralizing antibodies were investigated. As expected all intracellular viral markers were lower in aviremic as compared to viremic subjects, but in addition both elite and secondary controllers had lower levels of viral unspliced RNA in PBMC as compared to patients on HAART. *Ex vivo* cultivation of the virus from CD4+ T cells of SC consistently failed in one patient and showed delayed kinetics in the three others. Formal *in vitro* replication studies of these three viruses showed low to absent growth in two cases and a virus with normal fitness in the third case. T cell responses toward HIV peptides, evaluated in IFN-γ ELISPOT, revealed no significant differences in breadth, magnitude or avidity between SC and all other patient groups. Neither was there a difference in polyfunctionality of CD4+ or CD8+ T cells, as evaluated with intracellular cytokine staining. However, secondary and elite controllers showed higher proliferative responses to Gag and Pol peptides. SC also showed the highest level of autologous neutralizing antibodies. These data suggest that higher T cell proliferative responses and lower replication kinetics might be instrumental in secondary viral control in the absence of treatment.

## Introduction

Once infected with human immunodeficiency virus (HIV), the large majority of individuals are unable to control the virus. Exceptional patients, so-called elite controllers (EC), continue to have an “undetectable” plasma viral load (VL<50 copies/ml) without treatment [1]. Strong HIV-specific adaptive immunity, genetic factors and/or viral defects have been invoked to explain controller status. Elite controllers appear to harbor HIV-1 variants that encode Gag, Pol, Env and or Nef proteins that are less efficient than their counterparts of HIV-1 in typical/chronic progressors. Broad neutralizing antibodies or highly effective T cells with broad specificity are present in a number of EC [2]–[4]. Particular HLA B MHC antigens, including B27, B5701 and B58, are enriched in EC. This has been explained by the fact that CD8+ T cells restricted by these HLA molecules, recognize very conserved epitopes in Gag and that escape comes at a high fitness cost for the virus [5], [6].

Despite all described associations, it remains controversial which functional characteristics of T cell responses are important for control of viral replication and protection against disease progression. The following features have been suggested: strong proliferative T cell responses, preferential targeting of particular viral proteins (e.g. Gag better than Env) [7]; number of epitopes targeted or breadth [8], [9]; functional affinity of the T cell receptor or avidity; concomitant CD4+ and CD8+ T cell responses as well as “polyfunctionality” i.e. the simultaneous production of various cytokines such as IL-2 and TNF-α, besides IFN-γ, chemokines such as. MIP1-α and/or lytic factors such as perforin, granzymes and CD107a expression [10]–[13].

Most HIV-infected subjects ultimately become dependent on highly active antiretroviral therapy (HAART) for their survival. HAART has improved life expectancy and quality of life of all HIV-infected patients with progressive disease [14]. However, so far it is not possible to cure HIV infection mainly because latent reservoirs persist even in patients who are on effective combination treatment [15]. Cessation of HAART therefore results in viral rebound within days or weeks and pre-treatment VL levels are typically reached within one year after treatment interruption [16], [17].

In contrast to this general rule, we recently identified four exceptional subjects, who were first treated for progressive disease and then stopped HAART, but nevertheless kept their plasma virus undetectable for a long time. We have called these patients “secondary controllers” (SC). Similar phenomena have been described by others [18], [19], but the underlying mechanism responsible for this viral control remained unclear. Understanding the immune-viral interaction that could explain a SC status is important for the further development of immunotherapy, since the ultimate purpose of this type of intervention is to induce a “SC status” in all HAART patients.

To address this question, we compared five groups of HIV infected individuals, including SC, rebounders or “secondary non controllers” (SNC), patients under stable HAART, therapy-naïve progressors (TN), as well as EC. As possible “correlates of secondary protection” we quantified levels of intracellular viral markers, tried to cultivate the virus from the patients CD4+ T cells *ex vivo* and to formally study the replication kinetics of those viruses *in vitro*. In addition, we investigated several aspects of HIV-specific CD4+ and CD8+ T cell functions. The latter included *ex vivo* breadth, amplitude, avidity and polyfunctionality as well as proliferative responses upon triggering with HIV peptides.

## Materials and Methods

### Patients

Blood samples were obtained from HIV-1 infected individuals enrolled at the Institute of Tropical Medicine (ITM), Antwerp, Belgium. The study protocol was approved by the Institutional Review Board of ITM and by the Ethical Committee of the Antwerp University Hospital. All subjects gave written informed consent. Five groups of HIV-1 infected individuals were selected. (1) Secondary controllers kept their viral load (VL) below 1,000 copies/ml after cessation of HAART and showed a VL of <50 copies at month 6. (2) Secondary non controllers (SNC) had a VL >10,000 copies/ml 3 months after cessation of HAART. (3) HAART patients were on stable antiretroviral treatment with VL <50 copies/ml. (4) Non-controller therapy naive (TN) patients had a VL >10,000 copies/ml. (5) Elite controllers (EC) had a VL <50 copies/ml without any treatment for more than 3 years. All patients in the study were chronically infected with HIV-1 i.e. for at least 3 years.

### Peripheral blood mononuclear cells (PBMC) and plasma

Each patient enrolled in the study donated 3 times 100 ml blood with an interval of 3 months. Plasma samples obtained from these patients were frozen at −80°C in aliquots. PBMC were isolated on a Lymphoprep® density gradient (Lucron Bioproducts, Gennep, The Netherlands). From freshly prepared PBMC, CD4+ T cells were purified using positive magnetic selection (Miltenyi, Gladbach, Germany) and these were used to cultivate virus. At each time point 10 million PBMC were freeze-dried for extraction of DNA or RNA to perform HLA typing and measurement of intracellular viral markers. The rest of the PBMC were frozen as described elsewhere [20] and, after thawing, they were used to perform the immunological experiments.

### HLA typing and determination of CCR5Δ32

Intermediate to high resolution HLA class I-typing for the B locus was performed by the Red Cross Blood Transfusion Center at the Free University of Brussels in Jette, Belgium. The presence of CCR5Δ32 was determined for all SC, EC and SNC as described elsewhere [21].

### Amplification of *gag, pol and env* genes and HIV-1 subtyping

DNA was extracted from PBMC samples using commercial kits (Qiagen, Venlo, The Netherlands). Afterwards *gag, pol* and *env* were amplified as described elsewhere [22]. The sequences were assembled and edited using BioEdit. Subtype was determined by phylogenetic analysis. To determine whether HAART had induced important mutations that induced resistance, sequences were analyzed using HIV drug resistance database of Stanford University. Next, it was also analyzed whether important mutations were present in T cell epitopes therefore Los Alamos database was used.

### Quantitation of HIV-1 proviral DNA, unspliced and multiple spliced RNA

For quantitation of cellular HIV-1 RNA and DNA load, total cellular nucleic acids were extracted from ten million dried-frozen PBMC using the Boom procedure [23]. Levels of HIV-1 proviral (pr) DNA and both forms of cellular HIV-1 RNA, unspliced (us) RNA and multiple spliced (ms) RNA, were quantified by semi-nested real-time PCR, as described earlier [24]. The extracted DNA was directly subjected to two rounds of PCR amplification: a limited-cycle pre-amplification step and a real-time PCR step, using semi-nested primers. For RNA quantitation, the eluted RNA samples were first subjected to DNase treatment (DNA-free kit; Ambion Inc., Austin, Texas, USA), to remove HIV-1 prDNA, and subsequently to reverse transcription. For both usRNA and msRNA assays, two rounds of amplification with semi-nested primers were performed on the resultant cDNA. For all assays, no positive signals have been obtained from the no-template PCR controls, as well as from the PCR controls without the reverse transcription step (for RNA assays), which were included in the quantitation. The amounts of PBMC-derived HIV-1 DNA and RNA species were normalized to total cellular inputs, in a separate real-time PCR by using the detection kits for either β-actin or ribosomal RNA, respectively (both Applied Biosystems Inc., Foster City, California, USA) and expressed either as number of copies per 10^6^ PBMC for prDNA or as number of copies per µg total RNA for usRNA and msRNA. The sensitivity of all three assays was four copies per reaction, which translated into approximately 40 copies/10^6^ PBMC for the prDNA assay and 200 copies/µg total RNA for RNA assays (actual detection limits depend on the total cellular inputs of the PBMC samples). The linear range was at least five orders of magnitude. The sensitivity, reproducibility, and accuracy of these assays have been documented earlier [25].

### *Ex vivo* cultivation of virus and evaluation of *in vitro* replication capacity of the isolates

Purified CD4+ T-cells from all patients were cultivated with phytohemagglutinin (PHA 10 µg/ml) and interleukin (IL)-2 (10 U/ml, Gentauer) stimulated PBMC to produce autologous virus. In some cases (SC and EC) purified CD4+ T-cells were electroporated with siRNA against RcKp54 (square wave puls 500 V, 5 ms) and cultivated with CD8 depleted PHA and IL-2 stimulated PBMC. The relative replication capacity (fitness) of the *in vitro* cultivated viruses from three secondary controllers and four secondary non controllers was evaluated. To this end, PHA and IL-2 stimulated PBMC from 3 healthy donors were infected at multiplicity of infection (MOI) 10^−3^ with the viruses from the patients and BaL as a reference strain (NIH, Germantown, MD). Viral production was assessed by measuring HIV p24 concentrations by ELISA [26] over time in the supernatant.

### Assessment of HIV-specific T cell responses, screening ELISPOT

Interferon (IFN)-*γ* enzyme-linked immunospot (ELISPOT) assays (Diaclone, Besançon, France) were performed with thawed PBMC as described elsewhere [9]. In these assays 769 HIV-1 peptides, 15 amino acids long and overlapping by 11 amino acids obtained from NIH AIDS Research and Reference Reagent Program (Germantown, Maryland, USA) spanning the entire HIV proteome, were used. The number of spot-forming cells (SFC) per well was determined using an automated ELISPOT plate reader (AID, Strassburg, Germany). Responses were scored as positive if the number of SFC exceeded 50 per 10^6^ PBMCs and was at least 3 times higher than the mean SFC in the 3 negative control wells (containing PBMC but no peptides).

### Epitope Mapping

Mapping of T cell responses was performed by IFN-γ ELISPOT using a strategy based on a matrix of peptide pools as previously described [9], [27]. The 372 peptides spanning HIV-1 Gag and Pol were divided into 370 pools of 10–11 peptides each. Each peptide was included into two different pools. The final concentration of each peptide was 2 µg/ml.

### Avidity

T cell avidity refers to activation threshold in response to defined concentrations of exogenous peptide. It was measured by pulsing PBMC in IFN-γ ELISPOT assay with fivefold serial peptide pool dilutions. Only those peptide pools were used that were previously positive in the screening assay. T cells were determined as high avidity when the two lowest concentrations (3.2 and 0.64 ng/ml) were positive in ELISPOT assay.

### Polyfunctionality of T cells

Thawed PBMC (1*10^6^ per experimental condition) were washed and stimulated in the presence of Brefeldin A (Becton Dickinson, Erembodegem, Belgium) with medium, the standardized mixture of Cytomegalovirus-, Epstein Bar Virus and Influenza (Flu) peptides (CEF, also donated by the NIH Reagent Program) as well as HIV Gag or Pol peptide pools always at a final concentration of 2 µg/ml. Only peptide pools positive in the screening IFN-γ ELISPOT assay were used. After stimulation for 6 hours, cells were stained for membrane markers using the following conjugated monoclonal antibodies: anti-CD3-PerCP-Cy5.5, anti-CD4-Alexa 700, anti-CD8-Pacific blue, and anti-CD69-APC-Cy7 (BD Biosciences). Next, cells were fixed and permeabilized (BD lyse/Fix and BD Perm 2 solutions, BD Biosciences) and intracellular cytokines were stained with the following directly conjugated antibodies: anti-CD107a-phycoerythrin (PE)-Cy5, anti-IFN-γ-FITC, anti-TNF-α-PE-Cy7, and anti-IL-2-PE. Polychromatic flowcytometric analysis was done using a Cyflow ML flow cytometer (Partec, Munster, Germany). Between 200,000 and 500,000 events were collected per sample. The events were subjected to a lymphocyte gate by FSC versus SSC plot. Following identification of CD8+ T cells (CD3+ CD8+) and CD4+ T cells (CD3+ CD8−), a gate was made for each respective function within the CD3+CD8+ T-cells and within the CD3+CD8− T-cells, by representing SSC in the X-axis versus cytokine function in the Y-axis. After the gate for each function were created, we used the Boolean gate platform to create the full array of possible combinations, equating 16 response patterns when testing 4 functions (FlowJo, Treestar). For more detailed explanation on this methodology see: http://www.flowjo.com/v765/en/boolean.html and http://www.flowjo.com/v8/html/boolean.html

### T cell proliferation

Lymphoproliferative responses were determined using the (^3^H) Thymidine incorporation assay, as previously described [28]. Briefly, thawed PBMC from the different time points were incubated in six plicate at 2×10^5^ cells/well in 96 flat bottom well plates (Nunclon, Bornem, Belgium) in 0,2 ml complete medium supplemented with 2,5% heat-inactivated pooled human serum (PHS, PAA, Pasching, Austria). Cells were cultured at 37°C in a humidified 5% CO_2_ incubator in absence or in presence of each peptide pool separately. Only peptide pools that were positive in IFN-γ ELISPOT screening were tested in the proliferation assay. Cell cultures were pulsed for 8 hours on day 6 with 1 µCi/well (3H) Thymidine (Perkin Elmer, Massachusetts, USA). Cells were harvested onto glass filters and 3H incorporation was measured using a TopCount scintillation counter (Perkin-Elmer). T cell proliferation was reported as stimulation index (SI), determined by dividing the mean counts per minute for the peptide-stimulated wells by the mean for non stimulated control wells.

### Env gp160 cloning and pseudovirus construction

To determine the neutralization susceptibility of viral variants from subjects enrolled in the study, single cycle pseudoviruses were generated. Full-length env gp160 was amplified from purified PBMC from the patients via nested PCR and cloned into an expression vector either pSV7d or pcDNA4/TO (Invitrogen BV, Groningen, The Netherlands). To investigate the neutralization capacity against heterologous variants following pseudoviruses were obtained from the NeutNet program: SF162 (subtype B), DU174 (subtype C), 92BR025 (subtype C) and 92UG024 (subtype D) [29]. Pseudoviruses were generated in a 24-well plate by transfection of human embryonic kidney (HEK) 293T cells (obtained from ATCC) with pNL4-3.LucR–E– (NIH AIDS Research and Reference reagent program) and one of the Env expressing plasmids, just mentioned [30]. After 48 hours supernatant was harvested and stored at −80°C. The titers of the pseudoviruses were determined on TZM-bl cells, expressing Luciferase under the control of an LTR promoter. Infection of TZMbl cells was quantified using SteadyLite (Perkin Elmer) as a substrate for Luciferase. Emitted relative light units (RLUs) were quantified on a LB 941 Berthold luminometer (Alabama, US) [31].

### Purification of IgG

Because also patients on HAART are included in the study, neutralization assays can only be performed with immunoglobulin (Ig)G isolated from plasma to avoid interference by drugs. To this end a commercial kit (SpinTrap, GE healthcare, The Netherlands) was used and manufacturer's instructions were followed.

### Neutralizing antibody assay

Neutralizing capacity of each patients IgG against autologous and heterologous viruses was measured by a reduction in luciferase gene expression after a single round of infection of TZMbl cells with pseudotyped viruses. The 50% inhibitory dose (ID50) was calculated as the reciprocal IgG dilution resulting in a 50% reduction of RLU compared to the virus control. [32].

### Statistical analysis

For cellular HIV-1 load, statistical analyses were performed on log10-transformed values. Undetectable values of cellular HIV-1 DNA and RNA were left-censored at the detection limit of corresponding assays. Statistical analysis and graphical presentations were performed using Prism (version 3.0, GraphPad). For comparison of multiple groups, significance levels were determined by Kruskal-Wallis tests; when two groups were compared, Mann-Whitney tests were used. All statistical tests were two-sided. P values <0.05 were considered statistically significant.

## Results

### Clinical characteristics of the study population

Within the HIV cohort of the Institute of Tropical Medicine of Antwerp, including over 1700 patients, we identified 160 subjects, excluding pregnant women, who had been successfully treated for progressive HIV infection with full HAART, but at some point decided to stop treatment against medical advice. Four patients, further referred to as secondary controllers (SC), either showed a limited and short-lived rebound (P1 and P3) or no viral rebound at all (P2 and P4) and all 4 kept their VL at “undetectable” level (<50 copies) during at least 6 months after effective therapy interruption. Importantly, antiretroviral drug levels were undetectable in those SC. Some clinical observations of the first two (P1 and P2) have been described in a separate case report [33]. In the present extensive study we aimed to identify genetic, viral and immune factors, associated with the SC status.

To that end, we compared SC with “secondary non-controllers” (SNC), who show a strong and sustained viral rebound after stopping HAART. However, the low-to-absent viral replication in the SC could itself influence immune function. Therefore two other groups with low VL were included: elite controllers (EC), controlling virus replication below 50 copies without HAART, and patients under successful HAART. In addition, we also considered a group of regular chronically infected therapy-naïve (TN) patients with high VL. In each of the four comparator groups, patients were included over a range of peripheral blood CD4+ T counts, as can be seen in Table 1. Most patients were infected with HIV-1 subtype B or the closely related subtype D, with the exception of SC P4 (A1) and HAART P17 (CRF-02). The EC and TN patients were all male, whereas the other groups included one or two women. The TN patients were on average younger (35 years), as compared to the other groups (54.5 in EC; p<0.05 and 57 in HAART; p<0.005). The time since diagnosis was also lowest in the TN group (mean 5.25 years), whereas it was 9.5 years in EC, 11.5 years in SC, 12.25 years in HAART (p<0.05) and 14.25 years in SNC (p<0.01) respectively. The duration on HAART was similar in the relevant groups, whereas the range of VL extended to higher levels in the SNC as compared to the TN.

**Table 1:** Patients characteristics at enrollment

Group	Patient	HLA B type	CCR5Δ32	HIV-1 subtype	Sex	Origin	Age (years)	Years since HIV diagnosis	Days on HAART	Virus load before HAART (copies/ml)	Virus load at intake (copies/ml)	CD4 T count at intake (cells/µl)
SC^a^	P1	B4101–5201	No	B	M	Belgium	65	20	6290	2530^f^	<50^h^	250
SC	P2	B1402–3501	No	D	F	Belgium	55	11	2092	44475	<50	1316
SC	P3	B4002–5701	No	B	M	Venezuela	36	4	UK^g^	UK^g^	<50	244
SC	P4	B3701–4402	no	A1	F	Belgium	37	11	2420	78170	<50	1486
EC^b^	P5	B3901–4402	heterozygous	B	M	Belgium	36	7	0		<50	633
EC	P6	B1501–5701	no	B	M	Belgium	62	13	0		<50	614
EC	P7	B3901–4001	heterozygous	B	M	Belgium	61	9	0		<50	437
EC	P8	B0801–B2705	no	B	M	Belgium	59	9	0		<50	675
SNC^c^	P9	B1302–4501	no	D	F	Congo	51	17	5259	<400^f^	216,000^h^	726
SNC	P10	B0702–B1401	no	B	M	Belgium	40	14	2964	<400^f^	233,000	361
SNC	P11	B4001–4403	heterozygous	B	M	Belgium	47	11	3451	145253	13,800	345
SNC	P12	B1501–3701	no	B	F	Belgium	39	15	4616	30450	50,000	732
TN^d^	P13	B3906–5701		B	M	Belgium	38	10	0		4,280	361
TN	P14	B0702–1401		B	M	Belgium	27	1	0		69,600	582
TN	P15	B1801–3501		B	M	Brazil	34	1	0		26,000	425
TN	P16	B0801–1801		B	M	Belgium	41	9	0		101,000	310
HAART^e^	P17	B4402–5501		CRF02_AG	F	Belgium	52	12	4294	170000	<50	707
HAART	P18	B5501–5701		B	M	Belgium	51	12	4259	142754	<50	973
HAART	P19	B0702–3501		B	M	Belgium	58	16	5987	<400 f	<50	806
HAART	P20	B0801–4403		B	M	Belgium	67	9	3409	267000	<50	438

a SC = secondary controller

b EC = elite controller

c SNC = secondary non-controller

d TN = therapy-naïve patients

e HAART = patients under highly active antiretroviral treatment

f Viral load under AZT

g UK: unknown: this patient had already started HAART in another center.

h in SC and SNC treatment was stopped for at least 6 months

### Selected host genetic factors do not differ amongst patient groups

First, we investigated whether some host genetic factors could be associated with the secondary controller status. HLA-B5701, known as over-represented in EC [5], was present in one EC, in one SC and one TN. In fact, none of the HLA-B alleles, associated with disease (non) progression presented a skewed distribution. We also evaluated the frequency of CCR5 polymorphisms in both controller groups and the SNC, because Δ32 heterozygosity has been associated with slower disease progression [34]. Heterozygosity was present in two EC and one SNC; all other patients were homozygous for wild-type CCR5 (Table 1). The sequences of Gag, Pol and Env of the SC were analyzed using the Stanford database. No obvious defects in any of these genes and no CD8+ T cell associated escape mutations could be detected.

### Levels of intracellular viral markers discriminate the patient groups

**Figure 1:**
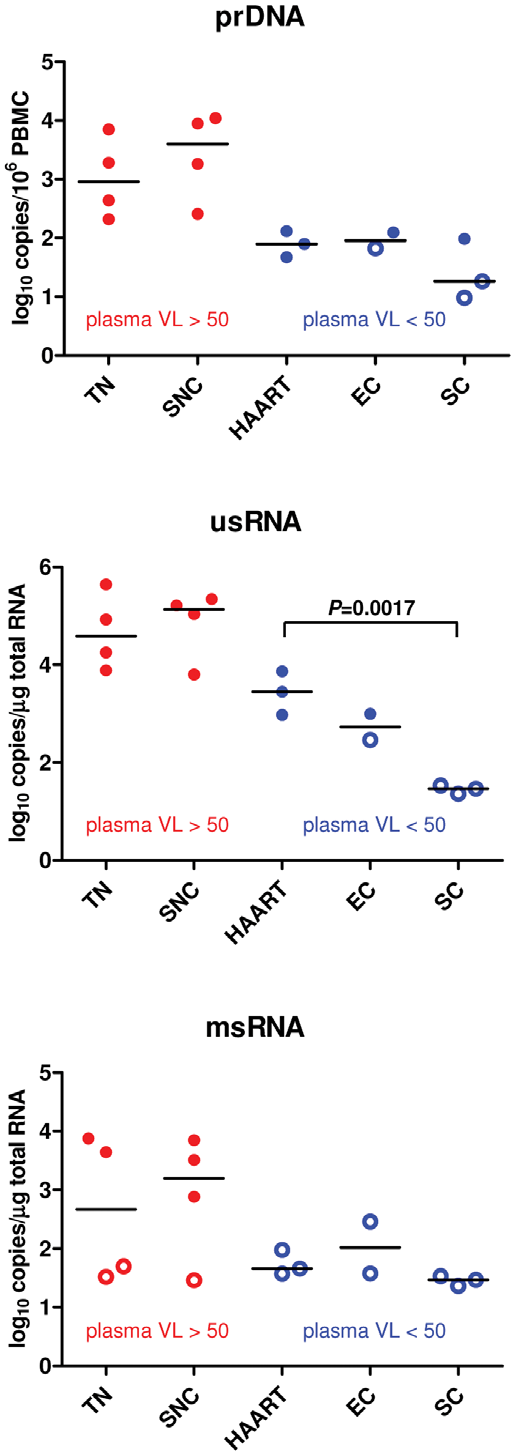
Levels of intracellular HIV-1 molecular markers in PBMC. Levels of proviral DNA (prDNA), unspliced RNA (usRNA), and multiply spliced RNA (msRNA) are shown. Patient groups with detectable plasma viremia (TN and SNC) are shown in red, and those with undetectable plasma viremia (HAART-treated, EC, and SC) are shown in blue. Horizontal bars represent median values. Undetectable values, left-censored at the corresponding detection limits (which were different for every sample, as they depended on total cellular inputs in the real-time PCR reactions), are depicted by open circles. Individual prDNA and usRNA values are means of two independent measurements. The statistical significance of the comparison between usRNA levels of SC and HAART-treated patients was calculated by use of unpaired *T*-test.

In order to understand the molecular details of intracellular HIV-1 dynamics, levels of proviral (pr)DNA, unspliced (us)RNA and multiplespliced (ms)RNA, were quantified on PBMC extracts (Fig. 1). Due to the primers used this analysis was restricted to subtype B and D infected patients. Expression of usRNA and msRNA can be linked to productive infection, whereas the prDNA amounts reflect the size of the pool of latently infected cells [24], [25]. In TN and SNC, high levels of usRNA and proviral load were present and the amount of usRNA correlated with the proviral load, as expected. In the HAART-treated patients, all being on suppressive therapy for over 9 years, the level of msRNA remained under the detection limit, whereas usRNA and prDNA levels were clearly lower than in the untreated viremic groups (SNC and TN), but nonetheless detectable in all patients. From the two EC tested the levels of usRNA and prDNA were detectable in one patient (P5). Remarkably, about one year after this measurement, P5 lost his EC status, as his plasma VL rose from <50 to 522 copies/ml, while the other EC patient kept his VL under the detection limit. Finally, in all SC both usRNA and msRNA were below the detection limit (p<0.005 for comparison of usRNA levels between SC and HAART-treated patients) and prDNA was extremely low or undetectable. In conclusion, with regard to intracellular viral markers, SC clearly differed from the HAART-treated patients (usRNA), despite plasma VL being undetectable in both these groups.

### *Ex vivo* isolation and *in vitro* replication of viruses from secondary controllers

**Figure 2:**
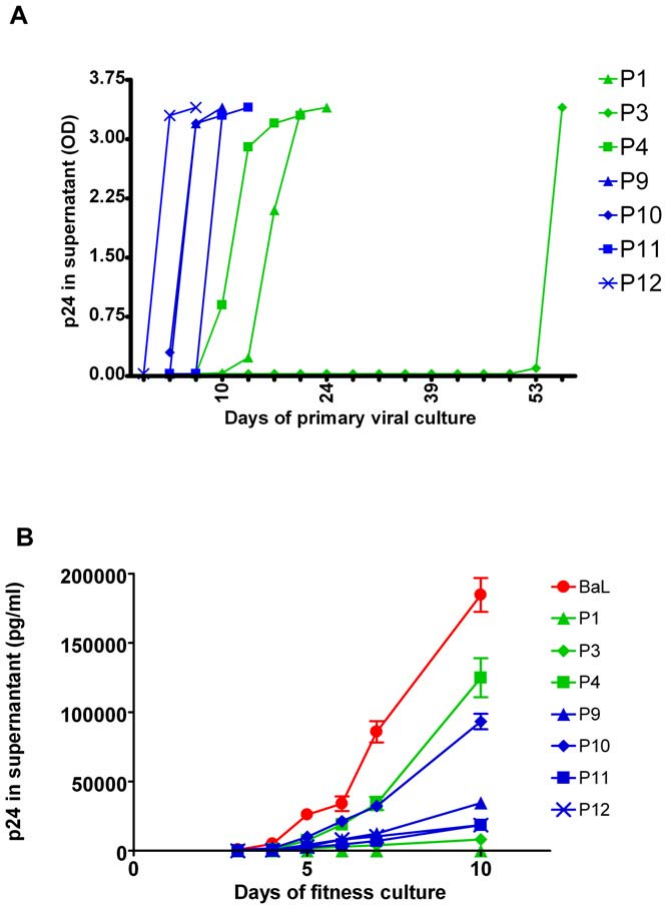
Replication characteristics of virus from secundary controllers. Ten million purified CD4+ T-cells from 4SC and 4SNC are cultivated with PHA and IL-2 stimulated PBMC. Panel A shows the virus replication in the primary culture (y-axis shows OD of p24 ELISA). These viruses were used in a secondary culture to evaluate fitness, by infecting new PHA and IL-2 stimulated PBMC at an equal MOI, with Ba-L virus as a reference. In panel B, the mean (and SEM) virus production in three donor PBMC is expressed as p24 in the supernatant. Green symbols are the SC; blue symbols are SNC and red symbol is BAL virus.

CD4+T cells from all patients were purified and used as such (first attempt) or after transfection with with siRNA against RcKp54 (subsequent attempts) to isolate the virus with activated donor PBMC. Virus could easily be cultivated from TN and SNC patients (always at the first attempt and within 1 week) and from HAART patients (first attempt and within 2 weeks). By contrast, despite two attempts, virus cultures from only 1 EC (P5) turned positive. Interestingly, this particular EC also had a detectable level of usRNA and was the one who subsequently lost his EC status. With regard to the SC, no virus could be cultured from P2, despite 3 attempts, while the others turned positive with delayed kinetics as compared to SNC (Fig. 2A): it took one attempt for P1 and P4, whereas only the second culture of P3 became positive after 53 days.

At first view these data suggest that of the three cultivable SC viruses, P3 is the least replication competent and P4 the fittest. However, the “cultivability” also depends on other factors (e.g. poviral load). Therefore, we formally compared replication kinetics of SC and SNC after adjusting their infectious titre to the same MOI and performed three independent “fitness” experiments in PBMC with the reference strain BaL as a positive control. As can be seen in Fig. 2B, the virus from P1 failed to grow and that from P3 was rather poorly growing, whereas P4 was slightly more replication competent than the viruses from SNC.

In summary, *in vivo* the proviral load of SC was very low, but sequencing failed to show gross abnormalities in their virus genes. Clearly, we have positive evidence in all SC patients that replication competent virus was present. For SC: P1, P3 and P4, the virus could be obtained in primary *ex vivo* culture patient's CD4+ T cells with delayed kinetics, as compared to SNC. Although the virus from P2 could not be cultivated, she showed a viral blip later in the evolution. In the secondary *in vitro* cultures, the virus from P4 was fully replication competent, whereas the viruses P1 and P3 showed a very low replication capacity. Taken together, these virological data apparently cannot fully explain the SC status and therefore a thorough immunological evaluation was performed as well.

### Breadth and magnitude of HIV-specific T cells responses is variable amongst patients in each group

**Figure 3:**
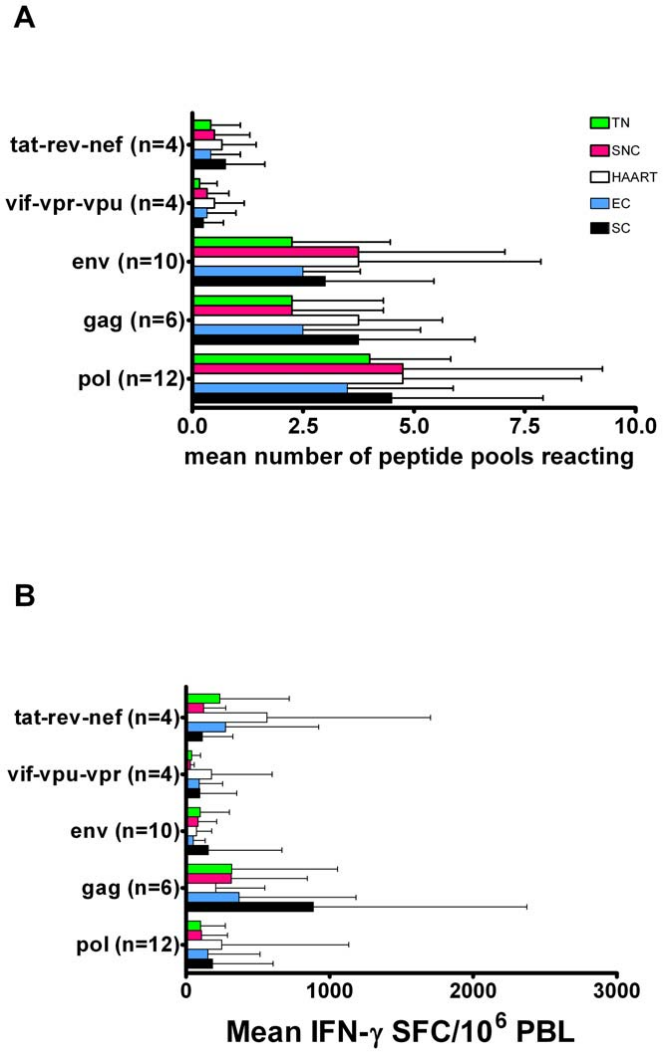
Breadth and magnitude of T cell responses against HIV peptides. (A) To evaluate T cell response breadth, PBMC from different patient groups were stimulated with peptide pools spanning the whole HIV genome. In the Y-axis the number of pools tested for each gene or gene group is indicated. In the X-axis the mean number (+SD) of peptide pools inducing positive ELISPOT responses in the HAART, TN, SNC, EC and SC patient groups is represented. (B) Based on the same dataset, the magnitude of the response was calculated as the mean number of IFN-γ spot forming cells (SFC) per million PBMC for each group of patients. Only peptide pools against which a positive response was measured, were included in this calculation. For each patient the SFC of positive peptide pools within each protein were cumulated and the mean (+SD) within each patient group is represented. No statistical differences were found.

In order to assess whether T cell responses to viral proteins could be associated with the viral status in our different patient groups, we first performed a comprehensive screening of *ex vivo* IFN-γ ELISPOT responses, using thawed PBMC, stimulated with peptide pools representing the whole HIV genome. Clearly, HIV-specific IFN-γ producing T cells were detected in all subjects. Comparing mean number of peptide pools recognized or breadth (Fig. 3A) and the magnitude of the responses within each viral protein or amplitude (Fig. 3B) did not reveal clear-cut differences between the patients groups, Remarkably, the mean amplitude of Gag responses was highest in SC, but individual variation was wide. With regard to breadth, the two SC P2 and P4, with high peripheral CD4+ T cells and no immediate viral blip after treatment cessation, had a very narrow response against Gag and Pol, reacting against 3 and 6 different peptides respectively while the ones with the low CD4+ T cell count and a small initial viral rebound (P1 and P3) had a very broad responses, reacting against more than 50 different peptides in Gag and Pol (Table 2). However, in the other groups there were also some patients with a narrow response and others with a broad response but this could not be correlated with CD4+ T cell count or viral load. Overall, these data show that there is heterogeneity in breadth and magnitude of these responses among HIV controllers as well as non-controllers.

**Table 2:** Summary of secondary controllers characteristics

		P1	P2	P3	P4
Clinical characteristics					
CD4 T count		Low	High	Low	High
Initial viral rebound		Yes	No	Yes	No
Late viral blips		No	One	No	Multiple
Viral parameters					
Intracellular prDNA		8	87	bdl^a^	ND^b^
Us/ms RNA		bdl	bdl	bdl	ND
Primary culture positive at day		13	Remained negative	53	10
Secondary fitness		No growth	Not possible	low	high
T cell responses to HIV peptides					
Breath (n° of pools recognized)		Broad	Narrow	Broad	Narrow
Magnitude (Σ SFC/10^6^ PBMC)					
- Tat rev nef		278	99	57.53	19
- Pol		278	18	369	77
- Vif vpr vpu		27	15	342	5
- Gag		918	65	2413	147
- Env		460	15	78	151
Polyfunctionality to Gag/Pol: (%)					
*° within CD3+CD8− T cells*					
	Single	85.71	69.14	93.11	82.45
	Double	12.47	29.28	6.72	16.45
	Triple	1.79	1.58	0.17	1.04
	Four	0.03	0	0	0.06
*° within CD3+CD8+ T cells*					
	Single	64.70	52.35	56.39	55.05
	Double	29.18	45.42	40.99	39.51
	Triple	5.71	1.77	2.31	5.06
	Four	0.40	0.46	0.3	0.37
Proliferation (SI)					
- Pol		5.5	1.1	3.3	1.2
- Gag		5.7	1.2	5.8	2.5

a : below detection limit

b : Not Done

### T cell avidity for Gag and Pol peptides is not different between patient groups

It was previously reported that CD8+ T cells that are associated with control of HIV replication, display high avidity, as evidenced by maintenance of T cell responses at very low (pg/ml) peptide concentrations [10]. Based on the results of the screening ELISPOT, using peptide pools in matrices, we deduced the epitopes in Gag and Pol, recognized by each patient separately. The selected peptides that were confirmed to be positive upon re-testing in a follow-up PBMC sample were used in a concentration range to evaluate avidity (Table S1). Only a few patients showed really high avidity responses in that ELISPOT remained positive at the lowest concentrations used. However, these patients are scattered over the SC (P2), EC (P8), SNC (P9 and P12) as well as the HAART group (P17 and P19). SC have no higher levels of strong avidity T cells as compared to the other groups and remarkably, also 2 out of 3 EC tested seemingly have no high avidity responses at all, suggesting that the presence of high avidity T cell responses to Gag and Pol is not associated with viral control in our SC and also not in our EC.

### Polyfunctionality of CD4+ and CD8+ T cells fails to discriminate viral controllers from non-controllers

**Figure 4:**
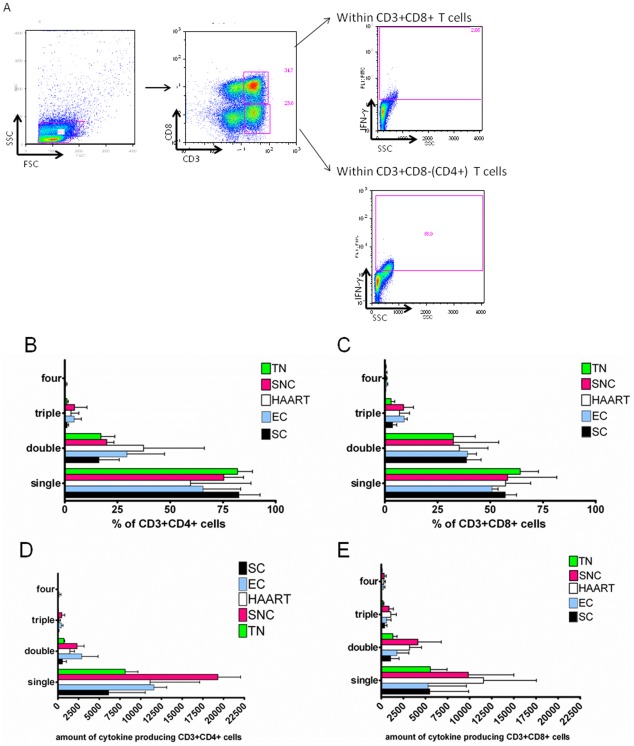
Evaluation of T cell polyfunctionality. PBMC from all study subjects were stimulated with peptide pools, selected for positive responses in the IFN-γ ELISPOT screening in each individual separately. In A the gating strategy for identification of multifunctional CD3+CD8+ and CD3+CD8− (CD4+) T cell responses is shown. After physical gating the mononuclear cells and excluding the dead cells, CD3+CD8+T-cells and CD3+CD8−(CD4+ T-cells) were identified. Within each of these populations the expression of IFN-γ, IL-2, TNF-a or CD107a was plotted against side scatter, to allow Boolean gating for the ultimate quantification of polyfunctionality. Polyfunctionality was analyzed using Flowjo by assessing the percentages of CD3+CD8− (hence CD4+) T cells (in B) and CD3+ CD8+ T cells (in C) that produce one, two, three or four cytokines. Percentages of co-expression for all selected peptide pools within one patient were summed and the mean+SD was calculated for each group. In D and E is the number of CD4+ T-cells respectively CD8+ T-cells that produce one, two, three or four cytokines presented. No statistical differences between groups were found.

Polyfunctionality, i.e. the capacity of CD4+ and/or CD8+ T cells to exhibit multiple effector functions simultaneously upon antigen encounter, is currently considered as one of the best correlate of T cell efficacy measurable directly *ex vivo* [35]. To evaluate this function, PBMC from each patient were stimulated with each peptide pool that showed positive SFC in the screening ELISPOT. The percentage of CD4+ and CD8+ T cells group that expressed individual and combined effector molecules, including CD107a, TNF-α, IL-2 and IFN-γ was measured. The gating strategy is illustrated in Fig. 4A. No differences in expression of individual cytokines/effector molecules were found between the patient groups (data not shown). Remarkably, percentages (Fig. 4B, C) and absolute numbers (Fig. 4D, E) of either CD4+ and CD8+ T cells expressing one, two, three or four functions, did not differ between the patient groups. Clearly, in our hands, polyfunctionality failed to distinguish controllers from non-controllers.

### SC and EC show more T cell proliferation towards Gag and Pol

**Figure 5:**
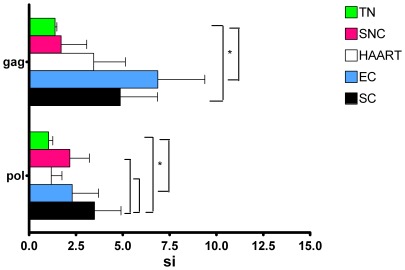
T cell proliferation against Gag and Pol peptides. PBMC from different patient groups were stimulated with Gag peptide pools 1–6 and Pol pools 1–12, spanning the entire Gag-Pol region. The mean stimulation index (SI)+SD for all 4 SC, EC, TN, HAART and SNC is shown. Statistical differences were measured using Mann-Whitney and Kruskall-Wallis test (P<0.05) and represented in the upper right insert.

Next, the proliferative capacity of T cells towards Gag and Pol peptide pools was evaluated. Mean proliferative responses in the various groups are shown in Fig. 5. Interestingly, SC had significantly more proliferative responses to Pol peptides than TN (p<0.01), SNC (p<0.0001) and HAART (p<0.01). Also T cells from both SC and EC had higher proliferative capacity tot Gag peptides than T cells from TN (p<0.05). As might be expected, the SC with the broadest and highest ELISPOT responses, also displayed the highest proliferative capacity (Table 2). Taken together, these results suggest that the capacity of T cells to proliferate is associated with control of virus replication.

### Neutralizing antibodies

**Figure 6:**
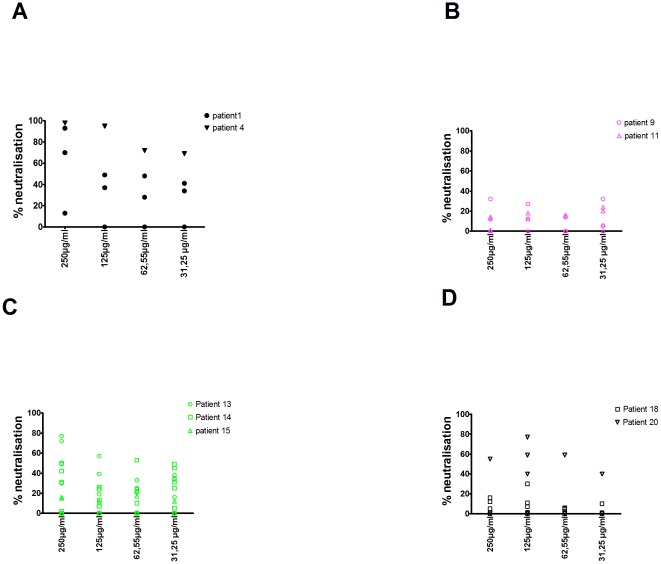
Autologous neutralizing antibodies. IgG was purified and Env was cloned from the same plasma sample in each patient. The neutralizing capacity of IgG was tested in the TZMbl assay, using single cycle chimeric pseudoviruses, containing the respective autologous Env. This assay could be performed for 2 SC (A), 2 SNC (B), 3 TN (C) and 2 HAART patients (D). Percent neutralization in Y-axis is shown over a concentration range of individual patient IgG, given in X-axis. Each patient is represented by one symbol and in many patients several autologous Env clones were tested.

We next quantified heterologous and autologous neutralizing antibodies in the different patients. To avoid the effect of anti-retroviral drugs (in HAART patients) and possible other plasma factors, IgG from the plasma of all patients was purified. IgG-mediated neutralizing capacity against 5 reference strains was low to absent in all patients and therefore no broad cross neutralizing antibodies were observed (data not shown). Autologous neutralization could be tested in two SC (Fig. 6A), two SNC (Fig. 6B), three TN (Fig. 6C) and two HAART patients (Fig. 6D). In elite controllers we were unable to amplify full gp160. When we accept the 50% level as significant at any IgG concentration, both SC patients P1 and P4 showed important autologous neutralization against at least one contemporaneous Env clone. However TN patient 13 and 14 and HAART patient 20 were also able to neutralize contemporaneous Env. The two SNC tested showed very low if any neutralization capacity. With regard to SC patient 1, sequence analysis of the obtained pseudoviruses clearly demonstrated the existence of at least 2 closely related viruses (data not shown). As shown in Fig. 6A, at the highest concentration used (250 µg/ml) the IgG neutralized one of the clones very potently (>90%), while the other pseudovirus was not neutralized (<50%). Interestingly also a recombinant virus having parts of both viruses could be amplified. Partial neutralization (65%) of this recombinant virus was obtained. Clearly, these data are limited, but they suggest that neutralizing antibodies might have a role in the SC status of this patient.

### Clinical follow-up

Samples for this study were taken in 2008. During the following 3 years, P1 and P3 kept an undetectable VL and their already low CD4 T counts tended to decrease further. Remarkably, P2 showed 1 viral blip of about 1000 copies and P4 showed multiple blips between 100 and 1000 copies/ml, but both kept their CD4 T count over 1000 cells/µL. Only P3 was put on HAART again, because of infectious complications.

## Discussion

In this study we describe a special group of patients, which we labeled “secondary controllers”. These HIV-1 infected individuals first showed clear-cut disease progression, were successfully treated for several years, stopped treatment and, unexpectedly, controlled their virus for many months or even years. This type of post-treatment interruption or “secondary” viral control might provide new clues to correlates or mechanisms of protection, which could be particularly useful to monitor immunotherapeutic strategies. We performed a comprehensive analysis of various host-viral interaction parameters. Remarkable, in common with equally aviremic but untreated elite controllers, the secondary controllers displayed very low level of HIV-1 usRNA and their virus was difficult to cultivate and had a lower fitness (except for SC P4). Relatively high T cell proliferation towards Gag and Pol peptides were also observed. In this respect, both controller groups behaved clearly different from viremic therapy-naïve progressors or secondary non-controllers as well as from aviremic patients under HAART. Interestingly, the two SC, from whom we could clone functional Env clones, showed the highest antibody-mediated autologous virus neutralization amongst all patients in various groups tested.

Surprisingly, we were unable to confirm correlations of either EC or SC status with other T cell functions, previously associated with viral control, including the breadth, the amplitude or the avidity of responses in ELISPOT. Nor was there any evidence of more polyfunctional T cell responses in either EC or SC group. Admittedly, there were some limitations to this study, including the small patient sample size and the use of frozen PBMC to assess polyfunctionality, which might explain why we failed to confirm previously reported correlations for EC.

Viral control and low setpoint have been attributed to specific HLA types (B57, B58, B27), with genome-wide associations studies ascribing 12% of viral setpoint variability to HLA related variation [36]–[38]. In this small study, no association was observed between carriage of HLA-B57 allele and viral control in either SC or EC. However, also other recent studies published about EC and viremic controllers failed to confirm these associations [39]. Likewise, specific CCR5Δ32 polymorphisms, previously associated with lower disease progression [34], were not present in SC, whereas 50% of EC were heterozygous for Δ32. Clearly, in our SC patients, the best known genetic markers could not explain controller status and therefore other mechanisms may be involved.

Previously it was demonstrated that the level of HIV-1 usRNA in PBMC, a marker of “active viral reservoir” (i.e. cells in which active viral RNA transcription and presumably production of viral antigens is ongoing), when measured in HAART-treated patients with undetectable plasma viremia, represents a strong predictive marker for the outcome of therapy [24], [40]. The level of the same marker, measured in untreated patients with stable plasma HIV-1 RNA load, was shown to significantly increase over time [40]. In the present study, most persons who spontaneously control virus (EC and SC) had undetectable usRNA levels and in one of the EC (Patient 5) with a measurable level of us RNA, plasma viremia emerged later. This is in contrast to HAART-treated patients, in all of whom usRNA was detectable despite undetectable plasma viremia. Interestingly, whereas usRNA was undetectable in all three SC tested, prDNA, a marker of total reservoir (latent and “active”) could still be measured in one of them. Taken together these data show that secondary control is associated with no or very low numbers of cells in which viral RNA and proteins are produced, but archival provirus may still be present. Conversely, the more “active reservoir” in all HAART-treated patients tested was sufficiently large to be detectable with our assays. This is because HAART only prevents infection of new cells, but not viral RNA transcription and protein production in the already infected cells. Therefore, natural HIV control, as opposed to the HAART-mediated control, may be exerted mainly through host immune mechanisms which eliminate cells expressing viral antigens (“active HIV reservoir”), possibly explaining why the size of this type of reservoir in natural controllers (SC and EC) was very low.

In our preliminary study, we provided evidence that the replication capacity of the endogenous virus in two of our SC was very low (35) and we were not able to culture the virus of the other two patients. In the mean time we were able to cultivate virus from 3 out of 4 SC and we performed a fitness analysis on the cultivated virus. The virus from P4 was easy to cultivate and showed a high replication capacity, the viruses from P1 and P3 were more difficult to cultivate and had a low replication capacity. The virus from P2 was not cultivable in our hands. Clearly, evidence of lower fitness was present in 3 out of 4 SC. Nevertheless, all four patients showed either an early (P1 and P3) or late viral blip(s) (P2 and P4) in vivo and therefore, again, a contribution of immune control seemed likely.

Many studies have focused on identifying the functional characteristics of CD8+ and CD4+ T cell mediated immune control [12], [41]–[44], including the ability of T cells to proliferate and secrete multiple cytokines [42], [43], [45]. Comparison of magnitudes and breadths of responses has shown that breadth of Gag-specific responses, as defined by IFN-γ release, was associated with antiviral activity and lower viral loads [46], whereas broad responses against Env were associated with faster disease progression [7]. We could not find any significant differences in breadth and magnitude of T cell responses between the different patient groups. Nevertheless, within the SC, two patients (P2 and P4) showed very narrow responses and the two others very broad responses, which seemed inversely correlated with the peripheral CD4+ T cell counts. However, all four SC could control virus replication indicating that neither breadth nor magnitude can explain their secondary controller status.

Avidity of T cells is another factor that could discriminate between virus control or lack thereof. Almeida et al. have shown that highly sensitive CD8+ T cells display potent HIV-suppressive activity [10], [47]. In contrast Harrari et al. have reported that HIV-specific CD8+ T cells with polyfunctional profiles are actually those that display lower rather than higher levels of antigen sensitivity [48]. In our study only 1 EC and 1 SC (P2) harbored high avidity T cells against Gag or Pol but this was certainly not significantly more as compared to the other groups. Therefore, we can conclude that the antigen sensitivity of T cells can not explain the SC status.

T cell polyfunctionality is often described as the ability of a cell to produce at least 3 cytokines and/or other effector molecules (chemokines, lytic factors…) and it has been associated with CD8+ T cell mediated virus control [12], [47], [49]. Moreover, comparison of polyfunctionality and proliferative capacity of immune responses between elite controllers and non-controllers has shown that the ability to control virus is associated with high numbers of HIV-specific CD8+ T cells that secrete both IL-2 and IFN-γ [12], [39], [49] and that these responses contribute to the ability of CD8+ T cells to inhibit virus replication *in vitro* [50]. Further on, CD8+ T cells from high Gag responders were significantly better able to proliferate [10]. In our study T cells from some SC and EC indeed showed a higher proliferative response upon stimulation with Gag and Pol peptides than TN, suggesting that HIV-specific T cell proliferative capacity may play a role in controlling virus replication. In contrast to previous studies, we could not prove that EC and SC had a higher level of polyfunctional T cells. However, the importance of polyfunctionality as a correlate of protection has also been questioned in the STEP vaccination trial, amongst others [51]–[54].

The role of neutralizing antibodies in the control of viral replication has been controversial. Some early studies have shown high titers of heterologous neutralizing antibodies in long-term-non progressors compared to progressors [55], [56]. However, more recently in EC heterologous neutralizing antibody titers were shown to be significantly lower than those observed in individuals with viremia [39], [57], [58]. In our study significant heterologous neutralizing antibody titers could not be detected in either HIV controllers or non-controller patients. With regard to antibodies, capable to neutralize the patient's own virus, levels of these “autologous neutralizing antibodies” reportedly are low in progressors [59], [60] and high in long term non progressors [61]. In another study on EC, however, the level of autologous neutralizing capacity was low [62]. The apparent discrepancy between LTNP (high autologous neutralization) and EC (low autologous neutralization) could indicate that a certain level of viremia (which can be present in LTNP but not in EC) is needed to drive and/or maintain the neutralizing antibodies. Here, relatively strong neutralizing activity against autologous virus was present in the two SC tested (P1 and P4) while it was lower or absent in the other groups. Unfortunately, we were not able to study autologous neutralization in our EC. Although our results in SC are suggestive, the contribution of autologous neutralizing antibodies to viral control needs more study.

In conclusion, despite the low number of patients studied, we were able to show that both EC and SC have a few viral and immune characteristics in common, which distinguish both types of controllers from non-controller groups, including aviremic HAART-treated patients. These include low levels of intracellular viral RNA, lower fitness and higher proliferative T cell responses towards Gag and Pol. Since these particular viral and T cell characteristics were absent, not only in viremic TN and SNC, but also in long-term aviremic HAART patients, it is tempting to speculate that they could be part of the mechanism by which SC and EC control the virus.

## Supporting Information

Table S1 **Supplementary data avidity of Gag (A) and Pol (B) specific T-cells in all patients.** ^A^: number of peptides of Gag and Pol that have reacted in the epitope mapping ELISPOT. ^B^: Number of deduced epitopes. This number can be equal or lower than in the “peptides reacting” column, due to the overlapping nature of the peptides format used i.e. a single epitope can be present in 2 or 3 overlapping peptides. ^C^: Number of peptides tested: if not enough cells were available to test all epitopes, only those were used that were predicted to be restricted by the patients HLA-type (Los Alamos database). ^D^: Number of epitope-containing peptides that showed no reaction, although they reacted in the first ELISPOT. As remark for this experiment blood from a later date was used, clearly illustrating that epitope recognition varies over time. ^E^: Number of epitope-containing peptides that provided a positive ELISPOT at each concentration.
(DOC)
