# Supplementary material for: Immune and Viral Correlates of “Secondary Viral Control” after Treatment Interruption in Chronically HIV-1 Infected Patients
Source: PLoS One. 2012 May 30;7(5):e37792. doi: 10.1371/journal.pone.0037792 (PMC3364270; doi:10.1371/journal.pone.0037792)
Supplement: Table S1 — Supplementary data avidity of Gag (A) and Pol (B) specific T-cells in all patients. A: number of peptides of Gag and Pol that have reacted in the epitope mapping ELISPOT. B: Number of deduced epitopes. This number can be equal or lower than in the “peptides reacting” column, due to the overlapping nature of the peptides format used i.e. a single epitope can be present in 2 or 3 overlapping peptides. C: Number of peptides tested: if not enough cells were available to test all epitopes, only those were used that were predicted to be restricted by the patients HLA-type (Los Alamos database). D: Number of epitope-containing peptides that showed no reaction, although they reacted in the first ELISPOT. As remark for this experiment blood from a later date was used, clearly illustrating that epitope recognition varies over time. E: Number of epitope-containing peptides that provided a positive ELISPOT at each concentration. (DOC) [file pone.0037792.s001.doc]

**Table S1**

1. **Gag**

| **Patient** | **Exp 1: Epitope mapping in Gag** | | **Exp 2: Avidity determination against Gag epitope-containing peptides** | | | | | | | |
| --- | --- | --- | --- | --- | --- | --- | --- | --- | --- | --- |
|  | **Peptides reacting^A^** | **Epitopes Deduced^B^** | **Peptides Tested^C^** | **Negative reaction at 2µg/ml^D^** | **Positive reaction at^E^** | | | | | |
|  |  |  |  |  | **2 µg/ml** | **400 ng/ml** | **80 ng/ml** | **16 ng/ml** | **3.2 ng/ml** | **0.64 ng/ml** |
| SC P1 | 34 | 9 | 9 | 6 | 3 | 2 | 1 | 1 | 0 | 0 |
| SC P2 | 2 | 2 | 2 | 0 | 2 | 1 | 0 | 0 | 0 | 0 |
| SC P3 | 40 | 13 | 13 | 4 | 9 | 8 | 8 | 4 | 1 | 1 |
| SC P4 | 2 | 2 | 2 | 0 | 2 | 2 | 1 | 1 | 0 | 0 |
| EC P5 | 0 | 0 | 0 |  |  |  |  |  |  |  |
| EC P6 | 19 | no cells | 0 |  |  |  |  |  |  |  |
| EC P7 | 5 | 3 | 3 | 3 | 0 | 0 | 0 | 0 | 0 | 0 |
| EC P8 | 1 | 1 | 1 | 0 | 1 | 1 | 1 | 1 | 1 | 0 |
| SNC P9 | 18 | 6 | 6 | 0 | 6 | 6 | 5 | 3 | 3 | 3 |
| SNC P10 | 0 | 0 | 0 |  |  |  |  |  |  |  |
| SNC P11 | 0 | 0 | 0 |  |  |  |  |  |  |  |
| SNC P12 | 16 | 6 | 2 | 0 | 2 | 2 | 1 | 1 | 1 | 1 |
| TN P13 | 24 | 9 | 9 | 4 | 5 | 1 | 1 | 0 | 0 | 0 |
| TN P14 | 11 | 4 | 4 | 2 | 2 | 1 | 1 | 1 | 0 | 0 |
| TN P15 | 0 | 0 | 0 |  |  |  |  |  |  |  |
| TN P16 | 4 | 3^c^ | 2 | 0 | 2 | 1 | 1 | 0 | 0 | 0 |
| HAART P17 | 32 | 9 | 9 | 6 | 3 | 2 | 2 | 2 | 2 | 2 |
| HAART P18 | 12 | 4 | 4 | 4 | 0 | 0 | 0 | 0 | 0 | 0 |
| HAART P19 | 17 | 5 | 5 | 3 | 2 | 1 | 1 | 0 | 0 | 0 |
| HAART P20 | 0 | 0 | 0 |  |  |  |  |  |  |  |

1. **Pol**

| **Patient** | **Exp 1: Epitope mapping in Po)** | | **Exp 2: Avidity determination against Pol epitope containing peptides** | | | | | | | |
| --- | --- | --- | --- | --- | --- | --- | --- | --- | --- | --- |
|  | **Peptides reacting^A^** | **Epitopes Deduced^b^** | **Peptides Tested^C^** | **Negative reaction at 2µg/ml^D^** | **Positive reaction at^E^** | | | | | |
|  |  |  |  |  | **2 µg/ml** | **400 ng/ml** | **80 ng/ml** | **16 ng/ml** | **3.2 ng/ml** | **0.64 ng/ml** |
| SC P1 | 28 | 10 | 10 | 3 | 7 | 6 | 3 | 2 | 0 | 0 |
| SC P2 | 1 | 1 | 1 | 1 | 0 | 0 | 0 | 0 | 0 | 0 |
| SC P3 | 25 | 11 | 11 | 5 | 6 | 5 | 5 | 3 | 0 | 0 |
| SC P4 | 4 | 2/2 | 2 | 0 | 2 | 0 | 0 | 0 | 0 | 0 |
| EC P5 | 10 | 6 | 6 | 4 | 2 | 2 | 0 | 0 | 0 | 0 |
| EC P6 | 9 | no cells | 0 |  |  |  |  |  |  |  |
| EC P7 | 7 | 6^c^ | 2 | 1 | 1 | 1 | 0 | 0 | 0 | 0 |
| EC P8 | 0 | 0 | 0 |  |  |  |  |  |  |  |
| SNC P9 | 37 | 8 | 8 | 5 | 3 | 2 | 2 | 2 | 2 | 2 |
| SNC P10 | 0 | 0 | 0 |  |  |  |  |  |  |  |
| SNC P11 | 2 | 2 | 2 | 2 | 0 | 0 | 0 | 0 | 0 | 0 |
| SNC P12 | 19 | 4 | 4 | 0 | 4 | 4 | 3 | 0 | 0 | 0 |
| TN P13 | 26 | 7 | 7 | 5 | 2 | 1 | 1 | 0 | 0 | 0 |
| TN P14 | 9 | 3 | 3 | 1 | 2 | 1 | 1 | 0 | 0 | 0 |
| TN P15 | 17 | 6 | 6 | 2 | 4 | 2 | 0 | 0 | 0 | 0 |
| TN P16 | 3 | 3^c^ | 1 | 1 | 0 | 0 | 0 | 0 | 0 | 0 |
| HAART P17 | 31 | 6 | 6 | 1 | 5 | 3 | 2 | 2 | 2 | 1 |
| HAART P18 | 4 | 3 | 3 | 3 | 0 | 0 | 0 | 0 | 0 | 0 |
| HAART P19 | 43 | 12 | 12 | 3 | 9 | 9 | 7 | 5 | 2 | 1 |
| HAART P20 | 2 | 2 | 2 | 2 | 0 | 0 | 0 | 0 | 0 | 0 |
